# Deciphering the Immunostimulatory Effects of β-Glucan on a Rainbow Trout (*Oncorhynchus mykiss*) Macrophage-like Cell Line (RTS11) by Whole Transcriptome Analysis

**DOI:** 10.3390/genes14061261

**Published:** 2023-06-14

**Authors:** Dean Porter, Shahmir Naseer, David Peggs, Charles McGurk, Samuel Allen Moore Martin

**Affiliations:** 1 Scottish Fish Immunology Research Centre, School of Biological Sciences, University of Aberdeen, Aberdeen AB24 2TZ, UK; 2 Skretting Aquaculture Innovation, Sjøhagen 3, 4016 Stavanger, Norway

**Keywords:** RTS11, RNA-seq, transcriptomics, β-glucan, immunomodulation, innate immune response

## Abstract

β-glucans are a commonly used immunostimulant/prebiotic in many aquaculture applications for boosting the immune status in fish. However, the method of action as an immunostimulant has not been fully deciphered. To determine the immunomodulatory effects of β-glucans on the innate immune response, we stimulated the rainbow trout spleen macrophage-like cell line (RTS11) with β-1,3/1,6-glucans for 4 h. This study uses a whole transcriptomic approach to analyse the immunomodulatory properties of β-glucans. Several proinflammatory pathways were found to be enriched after stimulation, demonstrating the immunomodulatory effects of β-glucan supplementation. Several pathways relating to responses to bacteria were also found to be enriched. This study clearly demonstrates the immunomodulatory effects of the supplementation of β-glucans within an aquaculture setting and further validates the use of cell lines as predictive models to interpret the responses caused by dietary intervention.

## 1. Introduction

With the global population rising, increased food sources are needed to match the demand for dietary protein sources [1]. Aquaculture is the fastest-growing protein source and has a lower carbon footprint per Kg protein produced compared to traditional terrestrial meat farming practices [2]. With this increased production, there are ongoing risks involved with disease outbreaks [3]. Many bacterial pathogens are controlled by vaccination, but where no vaccines are available, or for newly emerging pathogens, the antibiotics used may lead to antimicrobial resistance [4]. As a part of health management in aquaculture, functional feeds are routinely used with a desire to improve fish health and immune competence to enable better outcomes following pathogenic challenges during production. Functional feeds can contain several different components that can modulate the immune response such as prebiotics, probiotics, immunostimulants, and other micronutrients [5].

β-glucans are well-studied immunostimulants/prebiotics that offer the host improved protection against several diseases such as *Aeromonas salmonicida* and viral hemorrhagic septicemia virus (VHSV) [6,7,8,9,10]. β-glucans are complex polysaccharides, made up of repeating units of glucose linked by glycosidic bonds, and are sourced from the cell walls of plants, yeast, or fungi. β-glucan’s immunostimulatory properties are well characterised in fish, with β-glucan supplementation causing the upregulation of proinflammatory genes such as *IL-1β, TNFα,* and *COX-2* [7,10,11,12]. However, to date, there is little information regarding the mechanisms of action of these important molecules in fish [13,14]. Several studies have suggested there may be a slight improvement in immune tolerance after treating with β-glucans before bacterial challenges [6,10,15]. However, the relationship between β-glucans and immune tolerance has not yet been fully explored. Recent studies using whole transcriptome analysis in both rainbow trout (*Oncorhynchus mykiss*) [9] and common carp (*Cyprinus carpio*) [16] have identified key molecular pathways that are modulated by β-glucan supplementation. A recent study in rainbow trout examined the impact of a β-glucan diet on fish that were infected with *A. salmonicida,* demonstrating that several immunostimulatory pathways were upregulated to a greater extent in fish fed with the β-glucan-supplemented diet after infection, with complement and coagulation, PI3K-AKT signalling, platelet activation, and T-cell receptor signalling pathways all enriched in the fish fed the β-glucan-supplemented diet [9]. In carp head kidney macrophages, C-type lectin type 4 was suggested as a novel β-glucan receptor, potentially leading to the direct activation of the C-type lectin signalling pathway. Other pathways relating to the proinflammatory response; cytokine-cytokine receptor interactions, apoptosis, NOD-like receptor signalling pathway, and ECM–receptor interaction were also upregulated in this species [16].

Innate immune cells recognise pathogen-associated molecular patterns (PAMPS) through pathogen recognition receptors (PRRs) which include C-type lectins (CLRs) and Toll-like receptors (TLRs), which activate defence mechanisms. In humans, macrophages express the CLRs: macrophage inducible C-type “calcium-dependent” lectin, CLEC4E (Mincle), macrophage C-type lectin (MCL) (CLEC4D), macrophage mannose receptor (MMR, CD206), and Dectins 1 (CLEC7A) and 2 (CLEC6A) [17,18]. CLRs have been characterised in salmonids, with CLEC4T being highly expressed in monocytes/macrophages in rainbow trout [19]. A study in carp head kidney macrophages by Petit et al., 2019, identified two C-type lectin domain encoding genes—C-type lectin family 4 member C (*CLEC4C*) and salmon C-type lectin A (*SCLRA*)—as candidate receptors for β-glucan binding [16]. M1 macrophages are typically the first line of defence against intracellular pathogens and promote proinflammatory signalling [20]. As a result, they play a key role in the recognition of molecules in the salmonid gut-associated lymphoid tissue (GALT) and are found in high numbers in the salmonid intestine [20,21]. M2 macrophages are involved in parasite parasitic infections and tissue repair [20]. In salmonids, there is a lack of cell lines specific to an individual immune cell type [22]. Cellular models are needed to understand the mechanisms of action and provide a fast-predictive model of immune function upon stimulation with functional ingredients.

In this study, the direct effect on cells for β-glucan is examined using the RTS11 (rainbow trout spleen macrophage-like 11) cell line [23], which has been used in many studies to examine immune responses [24,25,26]. The RTS11 cell line was kindly donated as a gift to researchers at the Scottish Fish Immunology Research Centre and has been kept in liquid nitrogen (N_2_) for long-term storage. The RTS11 cell line is characterised as a monocyte/macrophage-like cell line due to the presence of two distinct cell populations: small non-adherent cells and larger granular cells that are thought to be macrophages [23]. Β-1,3/1,6-glucans (M-glucans) was used due to its known ability to modulate RTS11 cells [10,12] and its commercial importance within aquaculture as a source of an immunostimulant in functional diets. Previous studies by Ordas et al. have shown that RTS11 cells are responsive to β-glucan stimulation after 24 h from concentrations of 30 to 120 μg/mL after 24 h, whilst the RTgutGC cell line only demonstrated an innate immune response when stimulated with the 120 μg/mL concentration after 24 h. The previous study by Porter et al. demonstrates that the duration of stimulation with M-glucans on RTS11 is effective after 4 h in producing an innate immune response, with key innate immune cytokine genes (*interleukin-1β, interleukin-8, tumour necrosis factor-α*, and *serum amyloid A*) being significantly upregulated, and it is a timepoint that allows for comparisons to be made against primary gut leucocyte cultures. To investigate the whole transcriptional response to β-glucan, RNA-seq followed by gene set enrichment was carried out, which identified several proinflammatory and CLR signalling pathways enriched in the RTS11 cell line. This study shows that macrophages stimulated with β-glucans can trigger a distinct proinflammatory and antimicrobial profile within the innate immune response within macrophages.

## 2. Methodology

### 2.1. In Vitro Cell Culture

RTS11 cells were cultured in flasks (75 cm^2^) at 20 °C in growth media (Leibovitz L-15 media + 30% FBS + 1% Penicillin/Streptomycin (P/S)). RTS11 cells were grown up from stocks stored in liquid nitrogen which showed similar phenotypes as those described by Ganassin and Bols [23] and had a passage number of 11. Before the stimulation, RTS11 cells were collected in two parts; the first step was to collect the media containing detached cells, after which 5 mL of fresh growth media was added to the flask and was used to aspirate the attached cells from the flask; this was then added to the media containing the detached cells. The cell suspension was centrifuged at 500× *g* for 10 min at 4 °C before being resuspended in fresh stimulation media (Leibovitz L-15 media + 1% FBS + 1% P/S). Cell concentrations were adjusted to 5 × 10^5^ cells/mL before 1 mL was added to 24 well plates. Stimulants were added as described below.

### 2.2. Stimulation with β-Glucans

The cells were stimulated using 100 µg/mL of M-glucans (Biotec, Norway) for 4 h. M-glucans is a commercially available form of β-glucans comprising β-1,3/1,6-glucans from yeast. Fresh M-glucan stock solutions were made from a powdered version, suspended in L-15 media, and stored at 4 °C until use. Stock solutions were then added to wells containing cells diluted in media to give the working solutions. Unstimulated RTS11 cells were used as a negative control. All experiments were performed in a randomised design in 24-well plates with a single well per sample, and the samples were treated independently through the entire protocol (n = 4).

### 2.3. RNA Extraction

RNA was extracted from cells in individual wells using 750 µL of TRI Reagent (Sigma, UK), following the manufacturer’s instructions. The RNA pellet was washed using 80% ethanol, dissolved in RNase-free water, and stored at −80 °C until use. The quality control of the samples was determined by a nanodrop spectrophotometer for the quantity and Agilent bioanalyser 2100 to determine the RNA integrity, where samples were ensured to be above 7.0 RIN (9.5–9.8 in all samples) before being used for library generation and sequencing.

### 2.4. Transcriptional Analysis: RNA-seq

RNA samples were enriched for Poly A^+^ RNA and used to generate True seq libraries (library preparation and sequencing was performed by Novogene Ltd., Cambridge, UK; sequencing was performed on the Novaseq platform with paired end sequencing PE150. The samples were sequenced at a depth of 40 M reads per sample, with a Q30 score of above 94%. All raw sequences have been deposited in the ArrayExpress repository under accession number E-MTAB-12643. RNA-seq datasets were processed using the Nextflow (v21.04.0) [27] NFcore RNAseq pipeline (v3.2) [28], with the option ”aligner star_RSEM” [29,30] and the ENSEMBL Rapid Release genome and annotation for rainbow trout (USDA_OmykA_1.1 (GCA_013265735.3)). Raw reads were trimmed within the NFcore pipeline using Trim Galore, with ~0.2% of all base pairs being removed. Raw counts were generated using the subread feature_counts [31]. The bioinformatic quality control of mapped reads and raw counts was carried out using MultiQC through graphical representation [32]. There were 64,944 rainbow trout transcripts identified in total with ENSEMBL gene ID annotation after the NFcore pipeline. At the time of writing, the ENSEMBL rapid release genomes were not annotated; hence, further annotation of the genes was required. These genes were used as the input to perform the DESeq2 package (v3.15) in R (v4.1.0) [33] to determine differentially expressed transcripts (DEGs) compared to unstimulated controls. During DESeq2 analysis, one control sample (RTS11_04) was removed from further downstream analysis (n = 3 for control samples). All in silico-derived proteins were subjected to BlastP analysis against the ENSEMBL genes of Zebrafish and Humans to generate Human genome nomenclature committee IDs (HGNC IDs) (official gene symbols) for each protein-coding gene. Genes were considered differentially expressed if they met the following criteria: padj < 0.05; log2fold change ≥ 1 = upregulated or ≤ −1 = downregulated (Supplementary Table S1). For gene set enrichment, Gene Ontology (GO) analysis and Kyoto Encyclopaedia of Genes and Genomes (KEGG) pathway analysis were performed using DEGs using the web interface DAVID [34,35]. Only protein-coding genes with unique HGNC gene identifiers were subject to GO and KEGG pathway analysis. The GO terms and KEGG pathways predicted were based on the HGNCs inputted into DAVID without using expression values. Upregulated DEGs and downregulated DEGs were inputted into DAVID separately so that the effect on the pathways was able to be identified, as no expression values are used. The visualisation of DEGs was carried out using a principal component analysis plot and a volcano plot in R (v4.1.0) using the ggplot2 package (v3.3.6). For the visualisation of enriched GO terms, a dotplot was generated using ggplot2 (v3.3.6). For KEGG pathway analysis, the web service Pathview was used to visualise pathways with DEGs imposed on top [36].

## 3. Results

After sequencing, approximately 40 million paired-end reads were generated per sample (Min = 39.0 million reads, Max = 57.7 million reads). The Q20 and Q30 values of all samples were approximately 98% and 94%, respectively, and the GC content was approximately 49–51%. For whole transcription analysis, the ENSEMBL genomes for rainbow trout (USDA_OmykA_1.1 (GCA_013265735.3) had 48,326 unique ENSEMBL identifiers. At the time of writing, these identifiers were not fully annotated; therefore, BlastP was used to quantify the rainbow trout proteome against both the human and zebrafish proteomes. A total of 46,065 genes were annotated in rainbow trout, with 47,330 and 48,079 genes mapping to human and zebrafish in silico-derived proteomes, respectively. HGNC identifiers for all genes mapped to humans were manually extracted, with these identifiers being used for downstream gene set enrichment by gene ontology and KEGG pathway enrichment analysis. 

### 3.1. Identification of Gene Expression Patterns in RTS11

**Figure 1:**
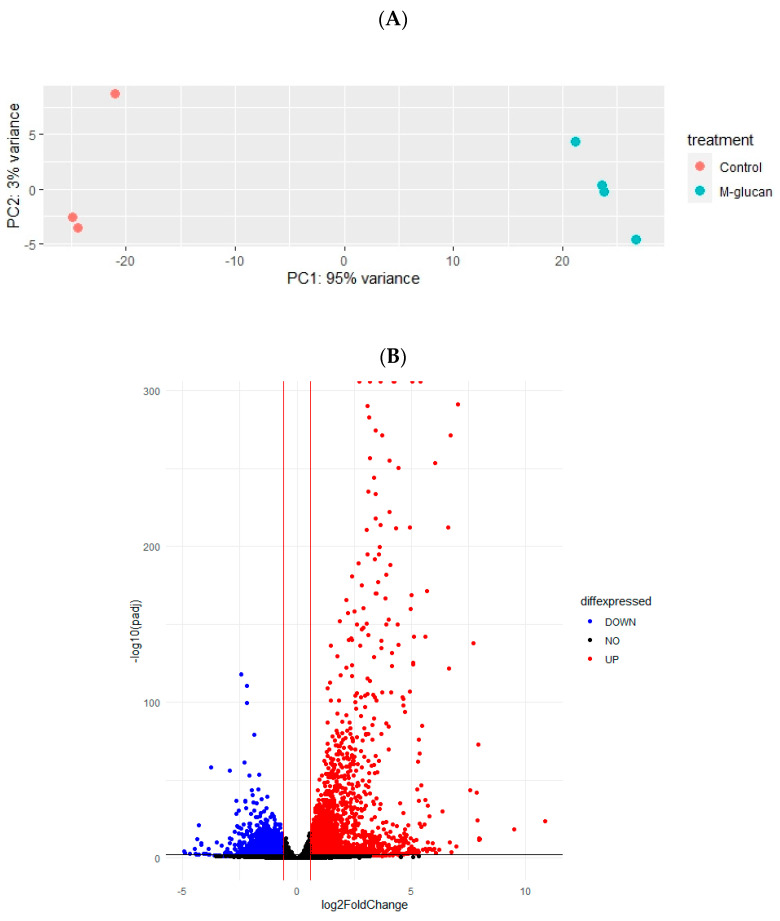
Identification of gene expression patterns. (**A**) Principal component analysis plot showing the distribution of RTS11 cells stimulated with M-glucans vs. control samples, where n = 4 and 3, respectively. (**B**) Volcano plot of all genes identifying differentially expressed genes (log2foldchange > 1, Padj < 0.05). Red represents an upregulated DEG, blue represents a downregulated DEG, whilst black represents a non-significantly expressed gene.

To visualise the transcriptome response to the β-glucan, a principal component analysis (PCA) plot was performed on RTS11 genes, which revealed the clustering of M-glucan-stimulated samples and unstimulated controls, showing a 95% variance between the two clusters (Figure 1A). A total of 28,644 genes were identified after filtering to remove genes where there were <10 counts. These genes were subjected to differential expression analysis (DESeq2), which resulted in the identification of 1635 genes with increased expression and 1057 genes with decreased expression following incubation with M-glucans (Figure 1B). The top 20 up- and downregulated protein-coding genes are presented in Table 1. The most upregulated gene encoded the *nuclear receptor subfamily 4 group A member 3* (*NR4A3*), being upregulated 10.8 log2 fold change, respectively. A key inflammatory chemokine gene, *CXCL2* (*C-X-C motif chemokine ligand 2*), and the proinflammatory gene, *TNFα* (*tumour necrosis factor-α*), were upregulated 6.6 and 6-log2 fold change, respectively. The most downregulated gene was the gene encoding for the *zinc finger BED-type containing 4* (*ZBED4*) protein, which was downregulated 4.6-log2 fold change. 

Of the RTS11 DEGs, 1635 upregulated and 1057 downregulated genes were represented by 1093 and 866 annotated protein coding genes annotated for HGNC identifiers. Prior to gene set enrichment, using these identifiers, HGNC duplicates were removed to ensure that the GO and KEGG pathway analyses were not affected by overrepresentation.

### 3.2. Gene Set Enrichment Using Gene Ontology

**Figure 2:**
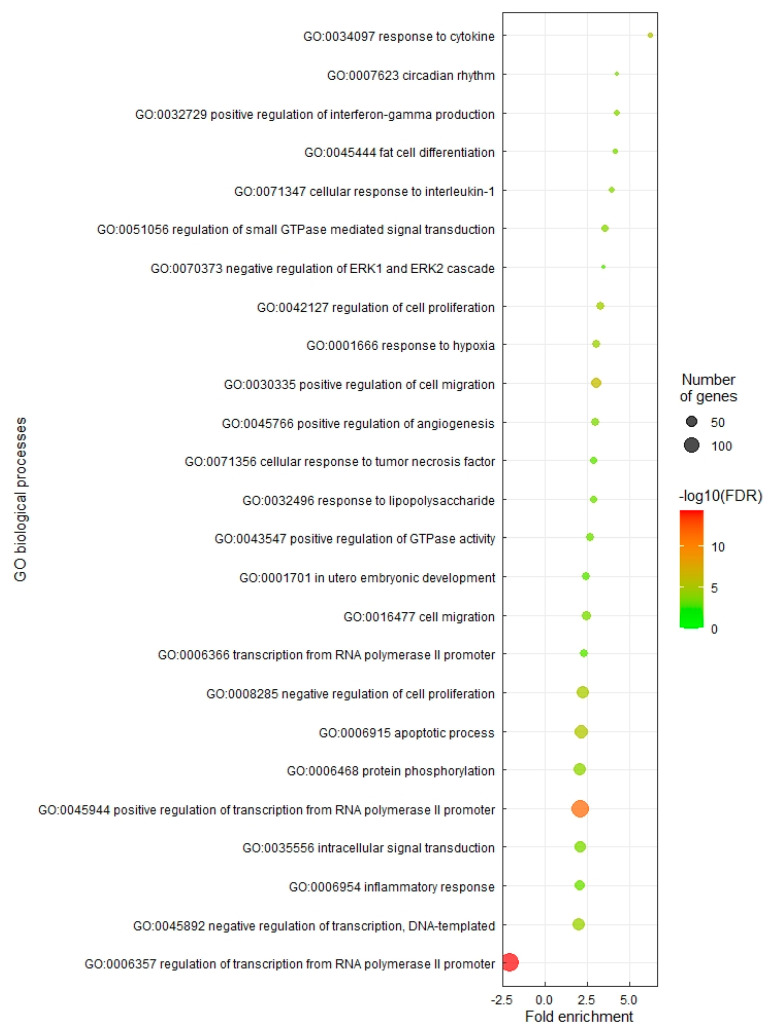
Gene ontology analysis of RTS11 stimulated with M-glucans for 4 h. GO terms have been filtered to show results with greater than 15 counts, greater than or less than twofold enrichment, and a Benjamini statistical score of less than 0.01. Colours represent −log10 (False Discovery Rate), with red being the highest, whilst the size of the dot represents the number of genes involved within each biological process.

The biological interpretation of transcriptomic changes in RTS11 following stimulation with M-glucans was performed using gene set enrichment. For GO enrichment, 25 Biological Processes terms were significantly enriched in RTS11 cells stimulated with M-glucans for 4 h (Figure 2). Many biological processes that were upregulated were related to the proinflammatory response; the GO:0034097 response to cytokine (6.25-fold change, 17/51 genes), the GO:0071347 cellular response to interleukin 1 (3.95-fold change, 19/90 genes); the GO:0071356 cellular response to tumour necrosis factor (TNF) (2.88-fold enrichment, 22/143 genes); the GO:0032496 response to lipopolysaccharide (2.88-fold enrichment, 24/156 genes); and the GO:0006954 inflammatory response (2.05-fold enrichment, 45/411 genes). For the downregulated DEGs, only a single GO biological process was significantly enriched: the GO term GO:0006357 regulation of transcription from RNA polymerase II promoters, which was enriched by 2.01-fold in this group. The GO terms relating to cellular components and molecular function can be found in Supplementary Tables S2 and S3. 

Gene ontology of the proinflammatory response to M-glucan supplementation: To further analyse the proinflammatory response being enriched, the two most upregulated pathways relating to the proinflammatory response, “response to cytokines” and “response to interleukin-1”, were selected to examine if there were differential responses between gene copies. For the GO “response to cytokines”, *Fos Proto-Oncogene (FOS), FOS ligand-1 (FOSL1),* and *Nuclear Factor Kappa B Subunit 2 (NFKB2)* show an upregulation of 4.65, 5.06, and 2.42-log2 fold change, respectively, as shown in Table 2. The GO term “response to interleukin-1” was enriched by 3.96-fold, with the key genes *CD40, prostaglandin I2 Synthase (PTGIS), Cys-Cys motif (C-C) chemokine ligand 20 (CCL20), CCL8*, and *CCL4* showing upregulations of 1.34, 2.85, 1.70, 2.65, and 3.31 log2 fold change, respectively, as shown in Table 3. Further analysis showing all rainbow trout paralogs of each significantly upregulated gene is found in Supplementary Table S4 (response to cytokine) and Table S5 (response to interleukin 1). 

### 3.3. KEGG Pathway Analysis

**Figure 3:**
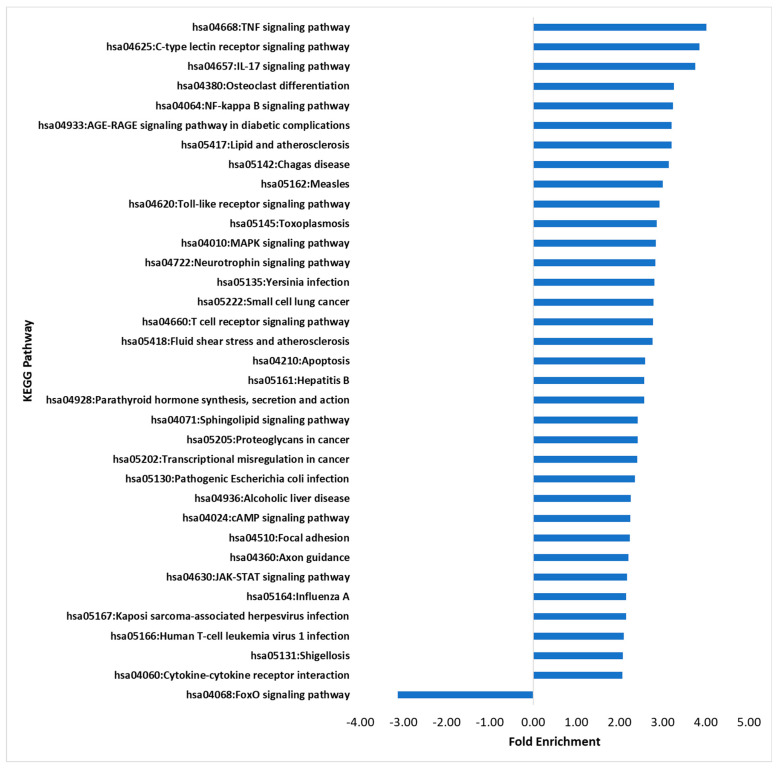
KEGG Pathway Analysis of RTS11 stimulated with M-glucans for 4 h. KEGG Pathway terms have been filtered to show results with greater than 15 counts, greater than or less than twofold enrichment, and a Benjamini statistical score of less than 0.05.

KEGG pathway analysis was carried out to detect significantly enriched pathways helping infer functional responses to the M-glucan. Nine pathways were enriched (Figure 3), with those relating to the immune response or inflammation being identified; these pathways included TNF (4.0-fold, 28/112 genes), IL-17 signalling (3.8-fold, 22/94 genes), T-cell receptor signalling (2.8-fold. 18/104 genes), and NF-κB signalling (3.2-fold, 21/104 genes). The pathways involved in pathogen recognition were enriched with the C-type lectin signalling (3.9-fold, 25/104 genes) and toll-like receptor signalling (2.93-fold, 19/104 genes), both being significantly upregulated. The pathways modulated in response to bacterial pathogens were also enriched, such as *Yersinia* infection (2.8-fold, 24/137 genes), pathogenic *Escherichia coli* infection (2.36-fold, 29/197 genes), and *Shigellosis* (2.1-fold, 32/247 genes). Only one pathway was found to be downregulated significantly, the FoxO signalling pathway (−3.13-fold, 16/131 genes).

To further assess the extent to which the immune response was being enriched by M-glucans, three specific pathways were selected. 

**Figure 4:**
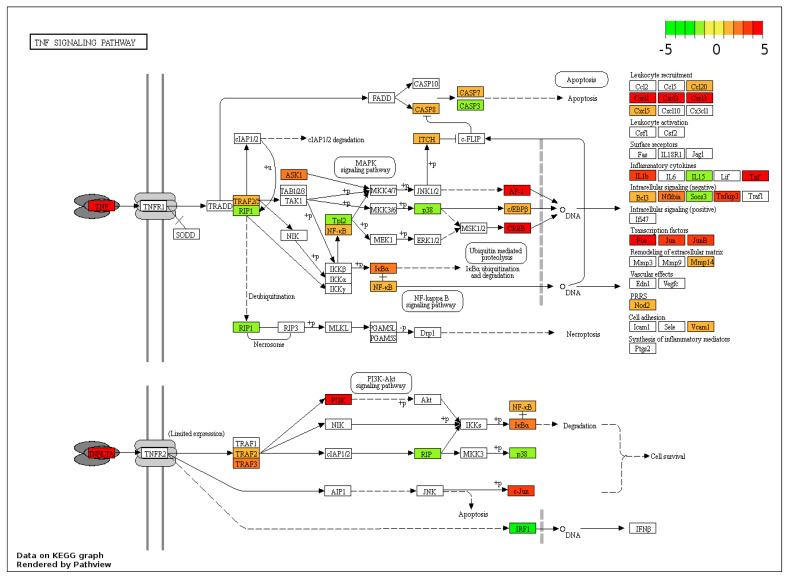
Regulation of the TNF signalling pathway by M-glucans. DEG datasets were mapped onto the pathway using Pathview. Genes regulated by M-glucans are shown in colour, with green showing downregulation and red showing upregulation. Black solid lines represent known molecular interaction or relation (▸: activating, |: inhibiting); dotted lines represent indirect links or unknown reactions, where +p, −p, +u, and −u represent phosphorylation, dephosphorylation, ubiquitination, and deubiquitination, respectively. The symbol O represents chemical compounds, DNA, or other molecules.

The “TNF signalling pathway” is a key anti-bacterial pathway induced by the chemokine tumour necrosis factor-α (TNFα), with downstream effects dependent on the cell type (Figure 4). The KEGG pathway map for the TNF signalling pathway was enriched by 4-fold, with 28 out of 112 genes showing significant upregulation, illustrating that many genes modulated within this pathway, including cytokines (*TNFα* and *interleukin 1-β (IL1β)*), genes involved with caspase (CASP) production (*CASP7* and *CASP8*), genes involved in leucocyte recruitment (*CCL20, Cys-X-Cys motif (CXC) chemokine ligand (CXCL)11, 12, 13, 14*, and *15*), and genes involved with transcription factors (*FOS*, *jun proto-oncogene (JUN)*, and *junb proto-oncogene (JUNB)*), were all upregulated. 

**Figure 5:**
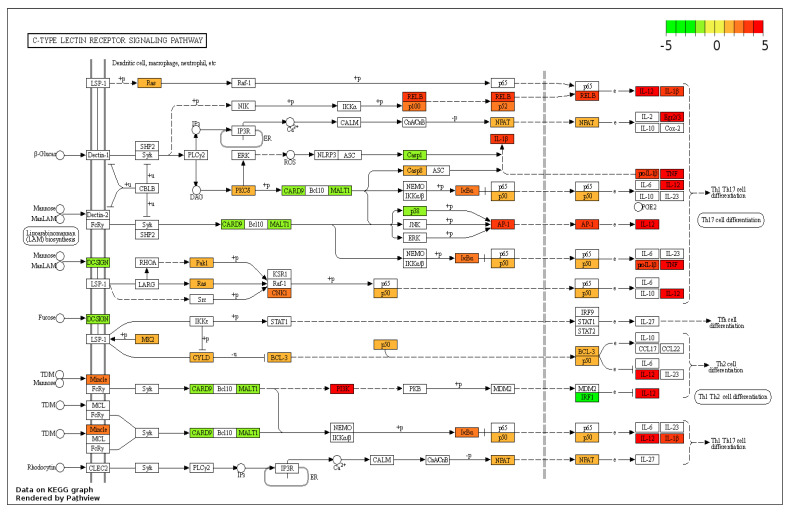
Regulation of the C-type lectin pathway by M-glucans. DEG datasets were mapped onto the pathway using Pathview. Genes regulated by M-glucans are shown in colour, with green showing downregulation and red showing upregulation. Black solid lines represent known molecular interaction or relation (▸: activating, |: inhibiting); dotted lines represent indirect links or unknown reactions, where +p, −p, +u, and −u represent phosphorylation, dephosphorylation, ubiquitination, and deubiquitination, respectively. The symbol O represents chemical compounds, DNA, or other molecules.

The KEGG pathway analysis of the C-type lectin signalling pathway in rainbow trout showed that 25/104 genes were upregulated with a 3.85-fold enrichment (Figure 5). The CLEC4E receptor was increased by 2.31-log2fold, with the additional downstream targets *interleukin 12 (IL12), TNFα,* and *IL1β* being upregulated. 

**Figure 6:**
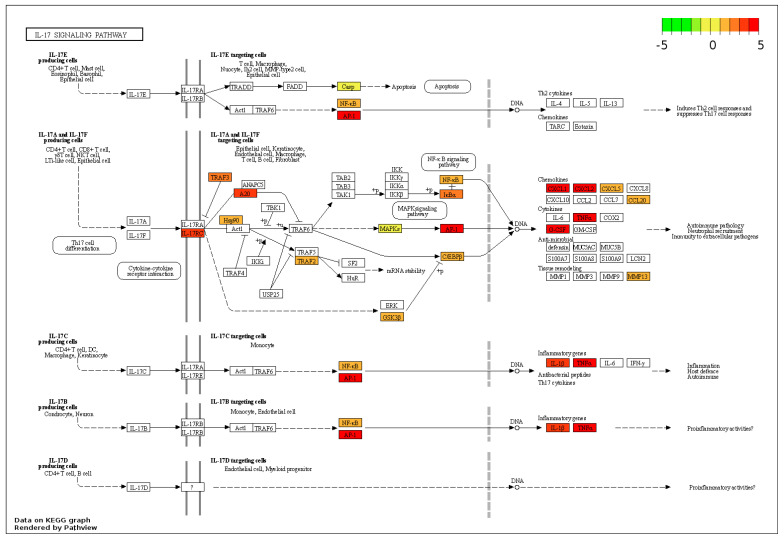
Regulation of the IL-17 signalling pathway by M-glucans. DEG datasets were mapped onto the pathway using Pathview. Genes regulated by M-glucans are shown in colour, with green showing downregulation and red showing upregulation. Black solid lines represent known molecular interaction or relation (▸: activating, |: inhibiting); dotted lines represent indirect links or unknown reactions, where +p, −p, +u, and −u represent phosphorylation, dephosphorylation, ubiquitination, and deubiquitination, respectively. The symbol O represents chemical compounds, DNA, or other molecules.

The other pathogen recognition receptor pathway to be upregulated was the toll-like receptor signalling pathway (Supplementary Figure S1). This pathway showed key downstream targets being upregulated: *IL-1β, TNFα, IL-12,* and *AP-1*; however, the receptors for this pathway showed no significant differences, except for *TLR5* and *TLR8*, which showed a 1.4 and 1.7 log2fold change downregulation. Immune pathways such as the IL-17 signalling pathway were also enriched by 3.75-fold, with the key molecules *NF-κB*, *heat shock protein 90 (HSP90), CXCL1, CXCL2,* and *TNFα* all being upregulated, as shown in Figure 6.

## 4. Discussion

This study further highlights the immune modulatory effect of β-glucans in rainbow trout by demonstrating the modulation of the innate immune system through the direct interaction with macrophage-like cells. This work expands on previous studies where targeted gene expression has been carried out by limited gene sets using real-time PCR in RTS11 cells [10,12]. Here, we clearly demonstrate that β-glucans cause the distinct immunomodulation of innate immune pathways relating to the proinflammatory response, most likely by binding to cellular receptors and resulting in multiple signalling pathways, rather than changes in the cellular metabolism. This is shown in both the gene ontology and KEGG pathway analysis of RTS11 samples. GO demonstrated that five overlapping biological processes were significantly upregulated, related to the proinflammatory response: the inflammatory response, response to lipopolysaccharide, cellular response to the tumour necrosis factor, cellular response to interleukin 1, and response to cytokines. This confirms previous studies in carp macrophages, which also showed differential expression in the same GO terms; inflammatory response and response to cytokines [16], showing that there are common responses across different fish species. Our results also complement previous in vivo experiments in the spleen where rainbow trout fed a β-glucan-supplemented diet and then infected with *A. salmonicida* showed KEGG enrichment in the complement signalling pathway, toll-like receptor signalling pathway, antigen processing and presentation, and T cell receptor signalling pathway [9]. There is a clear signature of inflammation, with specific immune transcripts (*IL1β, TNFα*, *IL8*) being upregulated that have also been observed in experiments with β-glucans before [7,8,10,11,12]. 

It is of interest that pathways related to innate cell surface receptors specifically, the C-type lectin signalling pathway and the toll-like receptor signalling pathways were strongly enriched by β-glucans in reports on both trout and carp [9,16]. In carp, the *C-type lectin 4C* (*CLEC4C*) and *Salmon C-type lectin receptor C* (*SCLRC)* were suggested to be potential candidate genes for a β-glucan receptor in carp [16]. *SCLR* genes, *SCLRA, SCLRB,* and *SCLRC,* were found to be upregulated after 7 days in the distal intestine of Atlantic salmon after being intubated with MacroGard^®^, a β-1,3/1,6-glucans from *Saccharomyces cerevisiae* [37]. In our analysis of RTS11 cells, both KEGG pathways relating to the TLR and CLR signalling pathways were enriched. Upon a closer examination of the TLR pathway, only downstream targets were significantly upregulated and not the receptors themselves, suggesting that a different pathway was causing the upregulation of certain cytokines. Within the CLR signalling pathway, both receptors and downstream targets were increased in expression. Of the significantly upregulated genes, two potential receptors were found to show significant upregulation: *C-type lectin domain family 4 member E* (*CLEC4E*/HGNC:14555) and *C-type lectin domain containing 17A* (*CLEC17A*/HGNC:34520). Interestingly, this is the same family of genes, C-type lectin family 4, that was seen to be potential candidate genes in the study by Petit et al., 2019. In human macrophages, CLEC4E has been shown to bind to trehalose-6,6′-dimycolate (TDM), a virulence factor found in the cell walls of *Mycobacterium tuberculosis* [17]. TDM is a disaccharide of two D-glucose molecules linked by a glycosidic bond, found predominantly in the α,α-1,1-trehalose form of the molecule [38]. TDM has some similarities to the molecular structure of β-glucans, a polysaccharide chain made from several D-glucose molecules linked by 1–3 β-glycosidic bonds, and, as such, may have similar binding receptors, further enhancing the likelihood of the CLEC4 family being a potential β-glucan receptor in rainbow trout. Further analysis of the C-type lectin family type 4 gene’s role in β-glucan responses will help determine the precise immunoregulatory roles of β-glucans.

The analysis of the GO and KEGG pathway analysis indicates that genes that are modulated by a bacterial response are also being driven by M-glucans. Here, the enrichment of GO terms, response to LPS, response to TNF, cellular response to IL1β, positive regulation of IFN-γ, response to cytokine, and inflammatory response are all highly enriched. These gene groups are all related to the proinflammatory response driven by bacterial PAMPs such as LPS or the upregulation of key cytokines, *TNF*, *IL-1β*, and *IFNγ*. KEGG pathway analysis demonstrates this further through the positive enrichment of the TNF signalling pathway, *Yersinia* infection, Pathogenic *Escherichia coli* infection, and *Shigellosis* pathways. Interestingly, these three bacterial pathways are all Gram-negative, facultatively anaerobic, non-motile bacteria belonging to the order *Enterobacterales*. *A. salmonicida* and *Vibrio anguillarum* are etiological agents for fish diseases and present threats to aquaculture. β-glucan supplementation was shown to improve the innate immune response to *Aeromonas* infection in rainbow trout [9,10], potentially by activating the antibacterial response that we show here, priming the fish to efficiently deal with the bacterial infection. The study by Petit et al., 2019a has demonstrated that the structure of the β-glucans used can modulate different pathways, with MacroGard^®^, a β-1,3/1,6-glucan, modulating more KEGG pathways compared to Curdlan, a β-1,3-glucan. 

This experiment only focuses on one concentration (100 μg/mL) and type of β-glucan (β-1,3/1,6-glucans), with further experiments needed to identify the effectiveness at lower concentrations or verify if other compositions of β-glucans would act more effectively. Nevertheless, the results of this study can provide a starting point for further research into β-glucans in aquaculture. In this study, “omics: technology was used to enhance the understanding of the immunomodulatory nature of β-glucans. Whilst “omic” data are useful in highlighting pathways of interest, further studies are needed to confirm the predictions provided by RNA-seq in this study. Further research is needed within aquaculture research to confirm the specific β-glucan recognition pathways, thought to be related to the C-type lectin signalling pathways in both the present study and that by Petit et al. [16], to allow β-glucans to be effectively used within feed production. 

This study further demonstrates the effectiveness of using cell culture models to establish baseline responses to functional ingredients. The use of cell culture models to replace in vivo experiments would help towards reducing the 3Rs and importantly provide an effective screening method for new functional ingredients that may benefit fish health. RTS11 has been used to establish innate immune responses due to its macrophage-like phenotype, which is found in the intestine of salmonids [21]. However, this cell line does not represent the cellular phenotype of the gut due to it being derived from the spleen, and as a cell line, it may only possess one or two clonal cell types. Alternatively, other intestinal cell lines such as the rainbow trout distal intestine (RTdi-MI), rainbow trout proximal intestine (RTpi-MI), or rainbow trout epithelial cell line (RTgutGC) could be used as novel models to identify immunomodulation in response to β-glucans [39,40]. Host immune responses are likely to be much more complex through the interaction of other antigen-presenting molecules, such as dendritic cells, interactions with the microbiome, and the respective short-chain fatty acids that are produced by β-glucan fermentation [41] and may overrepresent immune modulation due to the singular cell type. Further time points, such as 24 and 48 h, could also be tested to establish the responses of RTS11 at different phases of the innate immune response. The data shown here solely reflect rainbow trout, with further research needed to identify if the similar responses are expected in other commercial species. 

Conclusions: In the current study, β-glucans are shown to promote proinflammatory responses, with key proinflammatory pathways being upregulated, as demonstrated in many previous studies when using targeted gene expression [7,8,10,12]. RTS11 represents a macrophage-like cell line and provides a well-characterised, robust model for studying the implications of β-glucan supplementation on the innate immune response. RTS11 could be utilised, as in the present study, as a strong predictive model to understand immunomodulation and study immune responses to β-glucans alongside infection with *Aeromonas* or *Vibrio*. 

## Figures and Tables

**Table 1:** The top 20 up- or downregulated protein-coding genes detected against the rainbow trout genome after the DESeq2 analysis of RTS11 samples and a comparison between control and stimulated genes. Genes were selected based on Padj < 0.05 and those showing the greatest up- or downregulation in relation to log2foldchange.

HGNC ID	Gene Description	ENSEMBL Gene ID	log2FoldChange	Padj
NR4A3	nuclear receptor subfamily 4, group A, member 3	ENSOMYG00000016957	10.84	2.47 × 10 ^−24^
NR4A3	nuclear receptor subfamily 4, group A, member 3	ENSOMYG00000056982	9.50	2.48 × 10 ^−19^
EGR3	early growth response 3	ENSOMYG00000003397	8.01	1.31 × 10 ^−12^
DUSP2	dual specificity phosphatase 2	ENSOMYG00000015118	7.97	1.81 × 10 ^−13^
SYT11	synaptotagmin 11	ENSOMYG00000021392	7.95	1.85 × 10 ^−12^
EGR1	early growth response 1	ENSOMYG00000015637	7.93	1.08 × 10 ^−73^
OCSTAMP	osteoclast stimulatory transmembrane protein	ENSOMYG00000033187	7.90	6.59 × 10 ^−25^
INHBA	inhibin subunit β A	ENSOMYG00000053580	7.84	7.24 × 10 ^−43^
CNTF	ciliary neurotrophic factor	ENSOMYG00000033868	7.71	1.45 × 10^−138^
CCL4L2	C-C motif chemokine ligand 4 like 2	ENSOMYG00000026069	7.55	2.01 × 10^−44^
TRIO	trio Rho guanine nucleotide exchange factor	ENSOMYG00000037143	7.03	5.13 × 10^−292^
NAB2	NGFI-A binding protein 2 (EGR1 binding protein 2)	ENSOMYG00000012721	6.72	5.32 × 10^−272^
EGR3	early growth response 3	ENSOMYG00000045806	6.64	3.34 × 10^−122^
CXCL2	C-X-C motif chemokine ligand 6	ENSOMYG00000022444	6.60	6.8 × 10^−213^
SPRY4	sprouty RTK signalling antagonist 4	ENSOMYG00000031862	6.35	1.84 × 10^−30^
TNF	tumour necrosis factor a	ENSOMYG00000058346	6.04	4.29 × 10^−254^
AMER2	APC membrane recruitment protein 2	ENSOMYG00000061958	6.01	0.00000239
PRDM1	PR/SET domain 1	ENSOMYG00000011583	5.92	2.88 × 10^−10^
RNF208	ring finger protein 208	ENSOMYG00000009419	5.89	0.00000372
TMCC3	transmembrane and coiled-coil domain family 3	ENSOMYG00000005847	5.84	0.00000231
TIMM13	translocase of inner mitochondrial membrane 13	ENSOMYG00000009641	−3.48	0.030884
TESK2	Testis-associated actin remodelling kinase 2	ENSOMYG00000066315	−3.49	5.72 × 10^−11^
HHLA1	HERV-H LTR-associating 1	ENSOMYG00000045198	−3.50	0.019347
GPX2	glutathione peroxidase 2	ENSOMYG00000015731	−3.63	0.015558
CCNI	cyclin I	ENSOMYG00000006476	−3.75	4.85 × 10^−59^
ARMH4	Armadillo-like helical domain containing 4	ENSOMYG00000000520	−3.82	0.007779
C1orf21	chromosome 1 open reading frame 21	ENSOMYG00000003998	−3.85	0.010249
CD93	CD93 molecule	ENSOMYG00000014356	−3.86	1.01 × 10^−6^
ADORA2A	adenosine A2a receptor	ENSOMYG00000041298	−3.96	0.0024
IGKC	immunoglobulin kappa constant	ENSOMYG00000062277	−4.09	0.002041
TIRAP	TIR domain containing adaptor protein	ENSOMYG00000047353	−4.19	5.59 × 10^−9^
HES6	hes family bHLH transcription factor 6	ENSOMYG00000047424	−4.20	3.67 × 10^−10^
TMEM132D	transmembrane protein 132D	ENSOMYG00000046943	−4.28	0.004874
ZBTB45	zinc finger and BTB domain containing 45	ENSOMYG00000011021	−4.31	1.29 × 10^−21^
MYC	MYC proto-oncogene, bHLH transcription factor	ENSOMYG00000039293	−4.38	7.09 × 10^−13^
LHX4	LIM homeobox 4	ENSOMYG00000031792	−4.40	0.006305
PDXP	pyridoxal phosphatase	ENSOMYG00000015314	−4.45	0.001192
MFSD11	major facilitator superfamily domain containing 11	ENSOMYG00000003467	−4.46	9.54 × 10^−7^
RPE65	retinoid isomerohydrolase RPE65	ENSOMYG00000015503	−4.91	0.000398
ZBED4	zinc finger BED-type containing 4	ENSOMYG00000069563	−4.93	7.16 × 10^−5^

**Table 2:** Table showing the most differentially upregulated genes in the GO term GO:0034097-response to cytokine. The Log2foldchange displayed represents the most upregulated gene copy of the gene where paralogs are present (see Supplementary Table S2 for all copies of each gene).

HGNC	Gene Descprition	ENSEMBL_ID	log2foldchange	padj
TIMP3	TIMP Metallopeptidase Inhibitor 3	ENSOMYG00000011029	3.20	0
JUN	Jun Proto-Oncogene	ENSOMYG00000036861	4.06	9.9 × 10^−223^
RELB	RELB Proto-Oncogene	ENSOMYG00000021944	3.07	1.3 × 10^−195^
FOSL1	FOS ligand 1	ENSOMYG00000025915	5.07	6.6 × 10^−126^
NFKB2	Nuclear Factor Kappa B Subunit 2	ENSOMYG00000002188	2.42	2.5 × 10^−124^
FOS	Fos Proto-Oncogene	ENSOMYG00000029885	4.65	5.4 × 10^−103^
ALDH1A2	Aldehyde Dehydrogenase 1 Family Member A2	ENSOMYG00000046040	4.65	7.6 × 10^−99^
PML	PML Nuclear Body Scaffold	ENSOMYG00000027472	2.45	3.08 × 10^−42^
CD274	Programmed death-ligand 1	ENSOMYG00000032862	1.28	6.31 × 10^−42^
MAPKAPK2	MAPK Activated Protein Kinase 2	ENSOMYG00000032252	1.28	7.44 × 10^−36^
BCL2L1	BCL2 ligand 1	ENSOMYG00000043310	2.37	6.37 × 10^−32^
ITIH4	Inter-α-Trypsin Inhibitor Heavy Chain 4	ENSOMYG00000017068	2.40	8.07 × 10^−30^
SKIL	SKI-Like Proto-Oncogene	ENSOMYG00000042548	2.15	5.68 × 10^−21^
RARA	Retinoic Acid Receptor α	ENSOMYG00000041123	1.83	9.96 × 10^−15^
MAPKAPK3	MAPK Activated Protein Kinase 3	ENSOMYG00000042013	1.06	5.17 × 10^−8^
BCL2	BCL2 Apoptosis Regulator	ENSOMYG00000038826	1.48	1.71 × 10^−6^
SRF	Serum Response Factor	ENSOMYG00000038445	1.38	2.84 × 10^−5^

**Table 3:** Table showing the differentially upregulated genes expressed in the GO:0071347 Cellular response to Interleukin-1. The Log2foldchange displayed represents the most upregulated gene copy of the gene where paralogs are present (see supplementary Table S3 for all copies of each gene).

HGNC	Gene Description	ENSEMBL_ID	log2foldchange	padj
NFKB1	Nuclear Factor Kappa B Subunit 1	ENSOMYG00000040602	1.86	1.75 × 10^−152^
ZC3H12A	Zinc Finger CCCH-Type Containig 12A	ENSOMYG00000038385	3.32	2.34 × 10^−105^
CCL4	C-C Motif Chemokine Ligand 4	ENSOMYG00000008241	3.31	7.05 × 10^−86^
CCL3L1	C-C Motif Chemokine Ligand 3 Like 1	ENSOMYG00000026069	7.55	2.01 × 10^−44^
SOX9	SRY-Box Transcription Factor 9	ENSOMYG00000071421	3.48	6.63 × 10^−39^
CCL8	C-C Motif Chemokine Ligand 8	ENSOMYG00000002731	2.65	2.68 × 10^−32^
HIF1A	Hypoxia Inducible Factor 1 Subunit α	ENSOMYG00000040205	1.19	2.42 × 10^−31^
CEBPB	CCAAT Enhancer Binding Protein β	ENSOMYG00000019888	1.28	5.05 × 10^−28^
CD40	CD40 Molecule	ENSOMYG00000029243	1.34	1.92 × 10^−27^
MYC	MYC Proto-Oncogene	ENSOMYG00000041344	2.20	3.08 × 10^−23^
RC3H1	Ring Finger And CCCH-Type Domains 1	ENSOMYG00000029982	1.52	1.1 × 10^−21^
KLF2	KLF Transcription Factor 2	ENSOMYG00000039184	1.37	4.29 × 10^−17^
TANK	TRAF Family Member Associated NFKB Activator	ENSOMYG00000047138	1.18	1.83 × 10^−13^
GBP1	Guanylate Binding Protein 1	ENSOMYG00000036400	1.75	2.18 × 10^−11^
CCL20	C-C Motif Chemokine Ligand 20	ENSOMYG00000026966	1.70	0.0000010
ACOD1	Aconitate Decarboxylase 1	ENSOMYG00000028719	5.62	0.0000023
ADAMTS7	ADAM Metallopeptidase with Thrombospondin Type 1 Motif 7	ENSOMYG00000063119	1.17	0.00034
PTGIS	Prostaglandin I2 Synthase	ENSOMYG00000027171	2.85	0.02
NR1D1	Nuclear Receptor Subfamily 1 Group D Member 1	ENSOMYG00000024434	2.23	0.046

## Data Availability

The RNA-seq data have been deposited in the ArrayExpress repository (http://www.ebi.ac.uk/arrayexpress/) under accession number E-MTAB-12643.

## Supplementary Materials

The following supporting information can be downloaded at: https://www.mdpi.com/article/10.3390/genes14061261/s1, Supplementary Methods; Figure S1: Regulation of the Toll-like receptor signaling pathway by M-glucans; Table S1: All transcripts that were differentially expressed (padj <0.05, log2 fold change ≥ 1 = upregulated or ≤−1 = downregulated) and their relevant HGNC IDs; Table S2: Gene ontology analysis showing Cellular Component; Table S3—Gene ontology analysis showing Molecular Function; Table S4: All paralogs that are expressed in the GO:0034097 response to cytokine; Table S5: All paralogs that are expressed in the GO:0071347 cellular response to Interleukin-1.
