# Supplementary material for: Deciphering the Immunostimulatory Effects of β-Glucan on a Rainbow Trout (Oncorhynchus mykiss) Macrophage-like Cell Line (RTS11) by Whole Transcriptome Analysis
Source: Genes (Basel). 2023 Jun 14;14(6):1261. doi: 10.3390/genes14061261 (PMC10298332; doi:10.3390/genes14061261)
Supplement: Supplementary file 1 [file genes-14-01261-s001.zip › Porter_et_al_Supplementary_Figures_V3.pdf]

Supplementary Material

Supplementary Table S2 - Gene ontology analysis showing Cellular Component. GO terms have been filtered to show results with greater than 15 counts, greater than or less than 2-fold *enrichment, and a Benjamini statistical score of less than 0.01.*

Term	Count	Genes	Fold Enrichment	Benjamini Score
GO:0000785~chromatin	113	ATF1, BACH1, BACH2, ACTB, ETS2, FOXQ1, CCND2, MYC, SOX9, SOX7, JUNB, SOX4, MITF, RUNX3, HIC1, RUNX1, MYCL, DDIT3, ERF, ATF5, CLOCK, ATF3, ATF4, DLX3, TSHZ3, TSHZ2, CREM, FOXO3, HIF1A, EGR1, KLHDC3, JUN, EGR2, JUND, EGR3, BICRAL, PLK2, ESRRB, HMGA1, HMGA2, ESRRG, NFATC1, NR1D2, FOXN3, NR1D1, POU2F2, NFKB1, FOSL2, NFKB2, SMAD7, NR4A2, FOSL1, NR4A1, NR4A3, MAFB, SP4, BHLHE40, SNAI2, MAFK, NFE2L1, CSRNP1, GMEB1, GMEB2, CSRNP3, CHD7, WDR43, CIC, TEAD1, TGIF1, NCOA2, KLF11, KLF12, ZHX3, ARNT, PAX6, FOS, PROX1, ETV5, AEBP2, IRF1, TFEC, PAX9, RARA, AHRR, SMCR8, JDP2, CREB5, NFAT5, CEBPB, ZBTB46, SRF, FAAP24, RELB, ARNTL, NFIL3, ATOH8, MEF2D, IRX1, DMRT2, MBD2, NR1H4, KLF4, TBX5, KLF3, GRHL2, KLF2, PML, BATF, KLF7, KLF6, NEDD4, KLF9, BRDT	2.09	2.20E-11
GO:0005815~microtubule organizing center	19	ROCK2, NDE1, PDE4D, TRIOBP, TTC28, RELB, CYLD, PAK1, RASSF1, SMTN, SPECC1L, FAM110B, TACC1, TACC2, AJUBA, RAB11FIP4, ATF4, BCL2L1, NDEL1	2.32	4.04E-02
GO:0015629~actin cytoskeleton	32	CD274, MACF1, FAM107A, ARHGAP6, KALRN, TRIOBP, ACTB, LIMA1, ARSJ, FLNB, PDLIM5, GBP1, PDLIM7, SVIL, GAS2L3, DMTN, SHROOM4, MTSS2, MYO1D, MYO1E, SMTN, MYO1C, KANSL2, PALLD, CDC42EP4, SPECC1L, RARA, SPRY2, LCP1, BCAR1, MYO1F, DDR2	2.43	4.68E-04
GO:0043197~dendritic spine	21	PSD, RGS14, ZNF804A, SLA, ATP1A2, NR1D1, SORCS2, TANC2, PTK2, DNAJB1, GRM5, ABHD17B, SYT11, NEDD4, PDE4B, STRN, CRYAB, KCNJ2, SHANK1, LZTS3, CPEB4	2.45	1.33E-02
GO:0005912~adherens junction	23	FRMD4B, DLL1, PVR, SHROOM4, ACTB, SMAD7, VEGFA, CDH6, EFNB2, MYO1E, SH3BP1, CDC42EP4, DAG1, ITGA6, HMCN1, PDLIM5, AJUBA, FERMT2, S100A11, PDLIM7, CYTH1, VCL, LLGL2	2.57	3.50E-03

GO:0005925~focal adhesion	56	MDC1, RPL3, TGFB1I1, FAM107A, TNC, FHL2, FHL3, SLA, RND3, PVR, ACTB, TRIOBP, PLAU, CAPN5, DAG1, TNS4, TNS1, KLF11, ITGA2, ITGA1, SHROOM4, RHOB, MMP14, PALLD, NUMB, EVL, ITGA6, ARHGEF2, ITGA5, LCP1, ARHGEF7, AJUBA, PHLDB2, VCL, DDR2, GRB7, SDC4, PXN, NEDD9, SLC9A1, EFNB2, LIMA1, PAK1, GNA12, FLNB, PDLIM7, FZD1, SVIL, HSPA8, LIMK1, SPRY4, HMGA1, PTK2, FES, BCAR1, FERMT2	2.58	3.13E-08
GO:0005667~transcription factor complex	32	ATF1, NFAT5, CREM, HIF1A, ARNTL, EP300, SOX9, JUNB, TEAD1, SKIL, SOX4, TLE4, NCOA2, TLE3, JUN, JUND, HMGA1, ARNT, NFATC1, FOS, KLF4, TBX5, NR4A2, SKI, NR4A1, NR4A3, MAFB, DDIT3, RARA, ATF5, AJUBA, CLOCK	2.71	6.84E-05
GO:0005938~cell cortex	23	GPSM2, C2CD5, DENND2B, FRMPD1, PXN, MICAL3, NEDD9, TRAF2, FGF1, TRAK1, RND3, PTK2, RHOB, MYO1D, ITCH, NEDD4, FLNB, HMCN1, CCN2, ARHGEF7, FERMT2, STOX1, RAI14	2.74	1.38E-03
GO:0032587~ruffle membrane	15	PSD, MACF1, C2CD5, SPRY4, SH2D3C, FAM107A, MTSS2, PAK1, MYO1C, SPRY2, THEM4, PLEKHO1, LCP1, ARHGEF2, ITGA5	2.97	1.66E-02
GO:0001726~ruffle	16	ARHGAP18, FRMD4B, SAMSN1, LIMA1, PAK1, CLIP1, MYO1C, FGD6, PALLD, LCP1, ITGA5, ARHGEF7, S100A11, SH2B2, PDLIM7, BCAR1	3.10	6.86E-03
GO:0001725~stress fiber	15	PXN, FAM107A, FHL3, MST1R, SIPA1L3, SHROOM4, PTK2, LIMA1, PALLD, FLNB, LCP1, PDLIM5, FERMT2, SH2B2, PDLIM7	4.02	8.11E-04
GO:0090575~RNA polymerase II transcription factor complex	25	ATF1, CEBPB, HIF1A, BACH1, BACH2, NFIL3, MYB, JUNB, NCOA2, JUN, JUND, HMGA1, ARNT, FOS, BATF, FOSL2, FOSL1, MAFB, MAFG, DDIT3, RARA, ATF5, JDP2, ATF3, ATF4	4.04	8.14E-07

GO:0016604~nuclear body	30	MDC1, ZNF470, TNKS, PRDM15, TESK2, BMI1, HIF1A, CDC14A, IKZF4, SNAPC5, HMBOX1, LSM11, ZIC2, SNAPC2, HIVEP1, ECT2, ZNF300, CDT1, REXO1, CBX4, RMI1, TINF2, FLI1, POU6F1, PARP11, RNF169, GEMIN4, GEMIN7, HOXD3, PHPT1	-2.20	1.60E-02

Supplementary Table S3 - Gene ontology analysis showing Molecular Function. GO terms have been filtered to show results with greater than 15 counts, greater than or less than 2-fold enrichment, and a Benjamini statistical score of less than 0.01

Term	Count	Genes	Fold Enrichment	Benjamini Score
GO:0000785~chromatin	113	ATF1, BACH1, BACH2, ACTB, ETS2, FOXQ1, CCND2, MYC, SOX9, SOX7, JUNB, SOX4, MITF, RUNX3, HIC1, RUNX1, MYCL, DDIT3, ERF, ATF5, CLOCK, ATF3, ATF4, DLX3, TSHZ3, TSHZ2, CREM, FOXO3, HIF1A, EGR1, KLHDC3, JUN, EGR2, JUND, EGR3, BICRAL, PLK2, ESRRB, HMGA1, HMGA2, ESRRG, NFATC1, NR1D2, FOXN3, NR1D1, POU2F2, NFKB1, FOSL2, NFKB2, SMAD7, NR4A2, FOSL1, NR4A1, NR4A3, MAFB, SP4, BHLHE40, SNAI2, MAFK, NFE2L1, CSRNP1, GMEB1, GMEB2, CSRNP3, CHD7, WDR43, CIC, TEAD1, TGIF1, NCOA2, KLF11, KLF12, ZHX3, ARNT, PAX6, FOS, PROX1, ETV5, AEBP2, IRF1, TFEC, PAX9, RARA, AHRR, SMCR8, JDP2, CREB5, NFAT5, CEBPB, ZBTB46, SRF, FAAP24, RELB, ARNTL, NFIL3, ATOH8, MEF2D, IRX1, DMRT2, MBD2, NR1H4, KLF4, TBX5, KLF3, GRHL2, KLF2, PML, BATF, KLF7, KLF6, NEDD4, KLF9, BRDT	2.09	2.20E-11
GO:0005815~microtubule organizing center	19	ROCK2, NDE1, PDE4D, TRIOBP, TTC28, RELB, CYLD, PAK1, RASSF1, SMTN, SPECC1L, FAM110B, TACC1, TACC2, AJUBA, RAB11FIP4, ATF4, BCL2L1, NDEL1	2.32	4.04E-02
GO:0015629~actin cytoskeleton	32	CD274, MACF1, FAM107A, ARHGAP6, KALRN, TRIOBP, ACTB, LIMA1, ARSJ, FLNB, PDLIM5, GBP1, PDLIM7, SVIL, GAS2L3, DMTN, SHROOM4, MTSS2, MYO1D, MYO1E, SMTN, MYO1C, KANSL2, PALLD, CDC42EP4, SPECC1L, RARA, SPRY2, LCP1, BCAR1, MYO1F, DDR2	2.43	4.68E-04
GO:0043197~dendritic spine	21	PSD, RGS14, ZNF804A, SLA, ATP1A2, NR1D1, SORCS2, TANC2, PTK2, DNAJB1, GRM5, ABHD17B, SYT11, NEDD4, PDE4B, STRN, CRYAB, KCNJ2, SHANK1, LZTS3, CPEB4	2.45	1.33E-02
GO:0005912~adherens junction	23	FRMD4B, DLL1, PVR, SHROOM4, ACTB, SMAD7, VEGFA, CDH6, EFNB2, MYO1E, SH3BP1, CDC42EP4, DAG1, ITGA6, HMCN1, PDLIM5, AJUBA, FERMT2, S100A11, PDLIM7, CYTH1, VCL, LLGL2	2.57	3.50E-03

GO:0005925~focal adhesion	56	MDC1, RPL3, TGFB1I1, FAM107A, TNC, FHL2, FHL3, SLA, RND3, PVR, ACTB, TRIOBP, PLAU, CAPN5, DAG1, TNS4, TNS1, KLF11, ITGA2, ITGA1, SHROOM4, RHOB, MMP14, PALLD, NUMB, EVL, ITGA6, ARHGEF2, ITGA5, LCP1, ARHGEF7, AJUBA, PHLDB2, VCL, DDR2, GRB7, SDC4, PXN, NEDD9, SLC9A1, EFNB2, LIMA1, PAK1, GNA12, FLNB, PDLIM7, FZD1, SVIL, HSPA8, LIMK1, SPRY4, HMGA1, PTK2, FES, BCAR1, FERMT2	2.58	3.13E-08
GO:0005667~transcription factor complex	32	ATF1, NFAT5, CREM, HIF1A, ARNTL, EP300, SOX9, JUNB, TEAD1, SKIL, SOX4, TLE4, NCOA2, TLE3, JUN, JUND, HMGA1, ARNT, NFATC1, FOS, KLF4, TBX5, NR4A2, SKI, NR4A1, NR4A3, MAFB, DDIT3, RARA, ATF5, AJUBA, CLOCK	2.71	6.84E-05
GO:0005938~cell cortex	23	GPSM2, C2CD5, DENND2B, FRMPD1, PXN, MICAL3, NEDD9, TRAF2, FGF1, TRAK1, RND3, PTK2, RHOB, MYO1D, ITCH, NEDD4, FLNB, HMCN1, CCN2, ARHGEF7, FERMT2, STOX1, RAI14	2.74	1.38E-03
GO:0032587~ruffle membrane	15	PSD, MACF1, C2CD5, SPRY4, SH2D3C, FAM107A, MTSS2, PAK1, MYO1C, SPRY2, THEM4, PLEKHO1, LCP1, ARHGEF2, ITGA5	2.97	1.66E-02
GO:0001726~ruffle	16	ARHGAP18, FRMD4B, SAMSN1, LIMA1, PAK1, CLIP1, MYO1C, FGD6, PALLD, LCP1, ITGA5, ARHGEF7, S100A11, SH2B2, PDLIM7, BCAR1	3.10	6.86E-03
GO:0001725~stress fiber	15	PXN, FAM107A, FHL3, MST1R, SIPA1L3, SHROOM4, PTK2, LIMA1, PALLD, FLNB, LCP1, PDLIM5, FERMT2, SH2B2, PDLIM7	4.02	8.11E-04
GO:0090575~RNA polymerase II transcription factor complex	25	ATF1, CEBPB, HIF1A, BACH1, BACH2, NFIL3, MYB, JUNB, NCOA2, JUN, JUND, HMGA1, ARNT, FOS, BATF, FOSL2, FOSL1, MAFB, MAFG, DDIT3, RARA, ATF5, JDP2, ATF3, ATF4	4.04	8.14E-07

GO:0016604~nuclear body	30	MDC1, ZNF470, TNKS, PRDM15, TESK2, BMI1, HIF1A, CDC14A, IKZF4, SNAPC5, HMBOX1, LSM11, ZIC2, SNAPC2, HIVEP1, ECT2, ZNF300, CDT1, REXO1, CBX4, RMI1, TINF2, FLI1, POU6F1, PARP11, RNF169, GEMIN4, GEMIN7, HOXD3, PHPT1	-2.20	1.60E-02

Supplementary Table S4 - Table showing all paralogs that are expressed in the GO:0034097 response to cytokine

HGNC	ENSEMBL_ID	log2foldchange	padj
CD274	ENSOMYG00000032862	1.28	6.31E-42
	ENSOMYG00000013859	-0.36	1.75E-01
ITIH4	ENSOMYG00000017068	2.40	8.07E-30
JUN	ENSOMYG00000032401	3.64	0
	ENSOMYG00000036861	4.06	9.90E-223
	ENSOMYG00000018123	3.65	2.60E-214
	ENSOMYG00000025367	3.54	1.00E-177
SRF	ENSOMYG00000002699	1.07	1.40E-07
	ENSOMYG00000038445	1.38	2.84E-05
	ENSOMYG00000031869	0.91	1.22E-04
	ENSOMYG00000043815	0.60	6.80E-03
FOS	ENSOMYG00000002207	4.06	7.90E-189
	ENSOMYG00000029885	4.65	5.40E-103
PML	ENSOMYG00000027472	2.45	3.08E-42
	ENSOMYG00000027485	0.12	8.11E-01
RELB	ENSOMYG00000021944	3.07	1.30E-195
	ENSOMYG00000029822	2.39	1.60E-181
NFKB2	ENSOMYG00000040602	1.86	1.80E-152
	ENSOMYG00000002188	2.42	2.50E-124
FOSL1	ENSOMYG00000025915	5.07	6.60E-126
	ENSOMYG00000046245	4.93	1.10E-107
MAPKAPK3	ENSOMYG00000038077	0.85	2.27E-15
	ENSOMYG00000042013	1.06	5.17E-08
	ENSOMYG00000047837	-0.72	2.41E-01
ALDH1A2	ENSOMYG00000046040	4.65	7.60E-99
	ENSOMYG00000011624	1.57	3.61E-21
MAPKAPK2	ENSOMYG00000032252	1.28	7.44E-36
	ENSOMYG00000052035	0.85	2.66E-15
	ENSOMYG00000034995	0.70	5.13E-10
BCL2	ENSOMYG00000038826	1.48	1.71E-06
	ENSOMYG00000072108	-0.47	5.66E-01
RARA	ENSOMYG00000041123	1.83	9.96E-15
	ENSOMYG00000020321	0.28	1.08E-01
	ENSOMYG00000068443	0.60	3.60E-01
	ENSOMYG00000010146	-0.07	8.28E-01
	ENSOMYG00000010131	0.19	9.02E-01
	ENSOMYG00000014969	0.00	9.95E-01
TIMP3	ENSOMYG00000011029	3.20	0
	ENSOMYG00000050469	1.52	2.23E-13
SKIL	ENSOMYG00000042548	2.15	5.68E-21
	ENSOMYG00000029188	1.43	1.17E-06
BCL2L1	ENSOMYG00000043310	2.37	6.37E-32

	ENSOMYG00000022616	-1.97	4.56E-13
	ENSOMYG00000008424	1.05	8.70E-11

Supplementary Table S5 - Table showing all paralogs that are expressed in the GO:0071347 cellular response to Interleukin-1

HGNC	ENSEMBL_ID	log2foldchange	Padj
CEBPB	ENSOMYG00000019888	1.28	5.05E-28
	ENSOMYG00000005776	-0.31	1.05E-03
	ENSOMYG00000007878	0.99	1.51E-31
	ENSOMYG00000029243	1.34	1.92E-27
	ENSOMYG00000004142	-0.46	3.18E-04
CD40	ENSOMYG00000030358	0.47	8.35E-03
	ENSOMYG00000010299	-0.27	2.08E-01
	ENSOMYG00000004196	-0.39	3.98E-01
	ENSOMYG00000060044	0.01	9.89E-01
	ENSOMYG00000047645	1.01	2.80E-04
PTGIS	ENSOMYG00000036530	0.77	5.61E-03
	ENSOMYG00000027171	2.85	1.79E-02
	ENSOMYG00000045898	-0.66	3.10E-02
CCL3L1	ENSOMYG00000026069	7.55	2.01E-44
	ENSOMYG00000026966	1.70	1.01E-06
CCL20	ENSOMYG00000001207	-0.24	3.87E-01
	ENSOMYG00000041418	0.17	7.88E-01
ACOD1	ENSOMYG00000028719	5.62	2.29E-06
	ENSOMYG00000041722	0.82	6.77E-01
	ENSOMYG00000029982	1.52	1.10E-21
RC3H1	ENSOMYG00000046090	-0.65	8.39E-06
	ENSOMYG00000005000	-0.19	3.64E-01
	ENSOMYG00000008605	0.03	8.90E-01
NR1D1	ENSOMYG00000024434	2.23	4.58E-02
	ENSOMYG00000040205	1.19	2.42E-31
	ENSOMYG00000037394	-1.06	1.95E-12
HIF1A	ENSOMYG00000025188	0.63	1.42E-07
	ENSOMYG00000033186	0.41	5.34E-03
	ENSOMYG00000042767	-0.91	1.87E-01
	ENSOMYG00000047138	1.18	1.83E-13
TANK	ENSOMYG00000035513	0.67	3.88E-05
	ENSOMYG00000002066	0.27	1.56E-01
	ENSOMYG00000012726	0.31	3.21E-01
	ENSOMYG00000040602	1.86	1.75E-152
NFKB1	ENSOMYG00000006671	0.81	2.18E-16
	ENSOMYG00000030929	0.36	1.06E-03
	ENSOMYG00000039184	1.37	4.29E-17
KLF2	ENSOMYG00000002347	0.72	9.69E-02
	ENSOMYG00000018721	-0.58	6.71E-01
	ENSOMYG00000023459	-0.14	8.86E-01
CCL8	ENSOMYG00000002731	2.65	2.68E-32
	ENSOMYG00000002483	1.55	4.23E-56
	ENSOMYG00000041344	2.20	3.08E-23
MYC	ENSOMYG00000039293	-4.38	7.09E-13
	ENSOMYG00000020276	0.80	9.40E-13
	ENSOMYG00000005527	-0.22	6.75E-01
CCL4	ENSOMYG00000008241	3.31	7.05E-86
	ENSOMYG00000027049	-1.27	4.60E-01

	ENSOMYG00000038385	3.32	2.34E-105
	ENSOMYG00000058082	2.85	3.86E-18
ZC3H12A	ENSOMYG00000043954	-0.58	1.06E-01
	ENSOMYG00000012935	-1.45	2.64E-01
	ENSOMYG00000031296	0.30	3.23E-01
	ENSOMYG00000071421	3.48	6.63E-39
SOX9	ENSOMYG00000035344	3.32	6.47E-37
	ENSOMYG00000033159	2.34	2.67E-18
	ENSOMYG00000033469	1.64	6.18E-17
	ENSOMYG00000036400	1.75	2.18E-11
GBP1	ENSOMYG00000014414	-0.45	1.35E-02
	ENSOMYG00000075088	-0.27	1.27E-01
	ENSOMYG00000065961	-0.18	8.13E-01
	ENSOMYG00000033514	-0.06	8.64E-01
ADAMTS7	ENSOMYG00000063119	1.17	3.42E-04
	ENSOMYG00000071450	1.21	2.88E-01

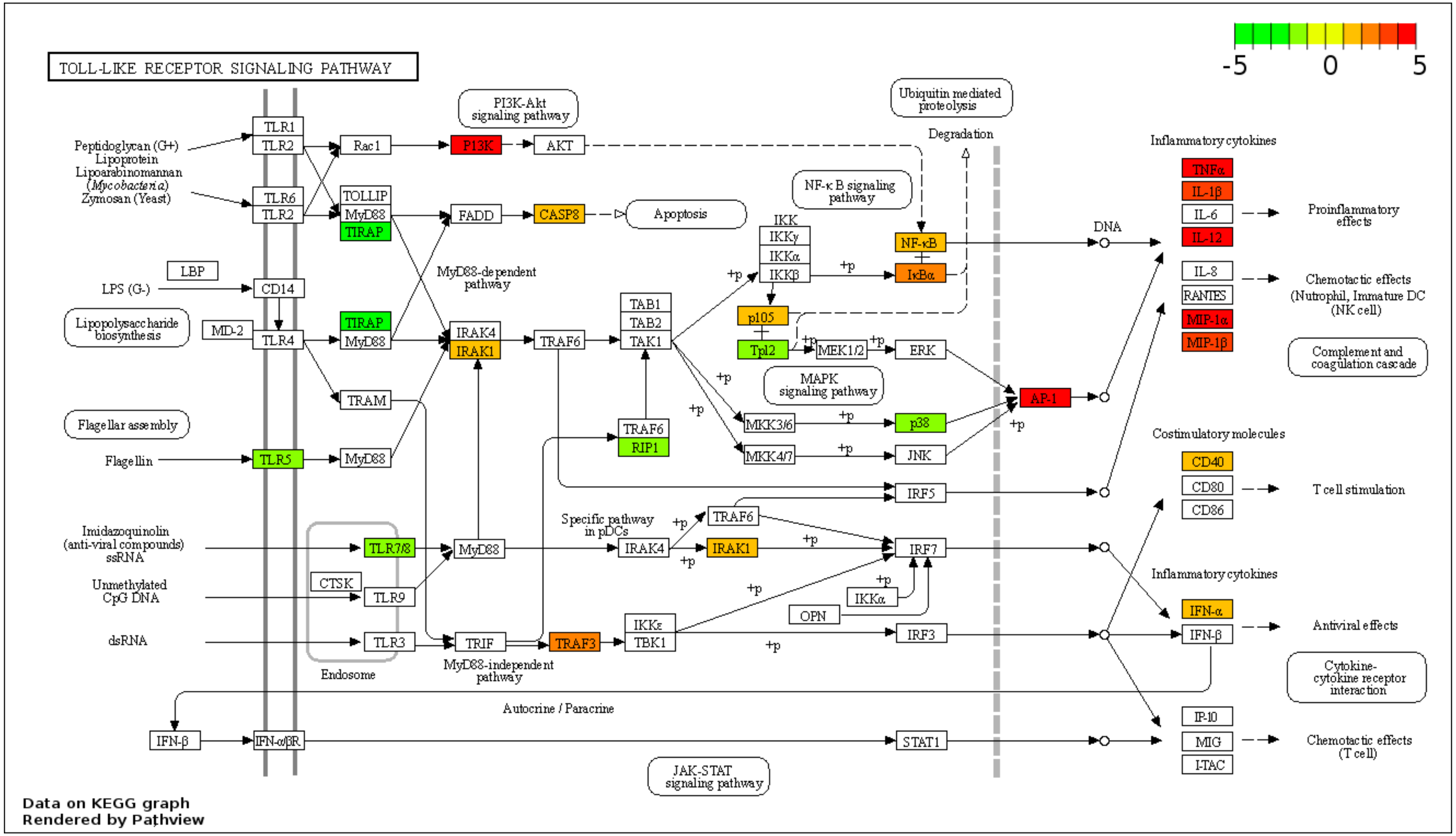

Supplementary Figure S1 - Regulation of the Toll-like receptor signaling pathway by M-glucans. DEG data sets were mapped on to the pathway using Pathview. Genes regulated by M-glucans are shown in colour with green showing downregulation and red showing upregulation. Black solid lines represent known molecular interaction or relation (‣: activating, |: inhibiting), dotted lines represent indirect links or unknown reactions, where +p, –p, +u, and –u represent phosphorylation, dephosphorylation, ubiquitination, and deubiquitination, respectively. The symbol O represents chemical compounds, DNA or other molecules.
